# Influence of Coal Gangue Powder on the Macroscopic Mechanical Properties and Microstructure of Recycled Aggregate Concrete

**DOI:** 10.1002/gch2.202300189

**Published:** 2023-10-03

**Authors:** Zhi Zhenli, Zhile Shu, Qihong Wu, Jiaxin Li, Haikuang Wu, Wenlong Chen, Xinhang Zeng

**Affiliations:** ^1^ School of Emergency Management Xihua University Chengdu 610039 China; ^2^ Sichuan Provincial Engineering Research Center of City Solid Waste Energy and Building Materials Conversion and Utilization Technology Chengdu 610106 China

**Keywords:** coal gangue powder, microstructural analysis, orthogonal experiments, recycled aggregate concrete

## Abstract

The construction and coal industries generate substantial industrial waste, including coal gangue and construction and demolition (C&D) waste, leading to environmental pollution and high disposal costs. Integrating recycled aggregates (RAs) and coal gangue powder (CGP) into concrete is an effective approach for waste management. However, CGP can affect the performance of traditional recycled concrete. This study primarily aims to optimize the utilization of RAs and CGP while maintaining concrete performance. They utilized orthogonal experimental designs and microscopic characterization techniques, including scanning electron microscopy (SEM), energy‐dispersive X‐ray spectroscopy (EDS), and X‐ray diffraction (XRD). Orthogonal experimental analysis indicated that with a water‐cement ratio (WCR) of 0.5 and replacement rates of 10% for CGP and 60% for RA, compressive and splitting tensile strengths reached 73.6% and 77.4% of ordinary C30 concrete, respectively. This mix proportion minimizes strength decline in coal gangue powder‐recycled aggregate concrete (CGP‐RAC) while maximizing recycled material replacement. Microscopic analysis revealed that CGP increased the Ca/Si ratio in cement paste, impeding hydration reactions, resulting in a looser internal structure and reduced concrete strength. These findings are anticipated to provide fresh theoretical insights for solid waste recycling and utilization.

## Graphical abstract

This study examined recycled concrete production using coarse recycled aggregates (RAs) to replace natural aggregates and a partial substitution of ordinary Portland cement with coal gangue powder (CGP). They identified the optimal CGP‐RA concrete mix ratio and analyzed microscopic factors affecting its performance decline. Their findings offer new insights for solid waste recycling.

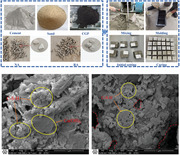

## 1 Introduction

Concrete, one of the most extensively utilized construction materials worldwide, has an average annual production of approximately one ton per person, resulting in a global output of ≈8 billion tons.^[^ ^1^ ^]^ However, due to its substantial depletion of natural resources, the sustainable utilization of concrete has garnered significant attention. Concrete itself constitutes 50−70% of total C&D waste globally.^[^ ^2^, ^3^, ^4^ ^]^ Furthermore, the rapid development of infrastructure and urbanization in countries such as China has led to a surge in the generation of construction and demolition waste.^[^ ^5^ ^]^ This mounting waste accumulation urgently necessitates sustainable approaches for the disposal of C&D waste to reduce the use of landfills.^[^ ^6^ ^]^ In 2018, China's construction industry alone accounted for an energy consumption equivalent to 1.1 billion tons of standard coal and emitted 2.72 billion tons of carbon dioxide during the production of building materials.^[^ ^7^ ^]^ Notably, cement production contributed to 130 million tons of coal equivalent in energy consumption and 11.1 billion tons of carbon emissions, representing 11.8% and 40.8% of the total energy consumption and carbon emissions during the construction material production phase, respectively.^[^ ^7^ ^]^ Consequently, achieving the sustainable utilization of concrete while reducing energy consumption and carbon emissions associated with cement production is crucial in attaining carbon peak and carbon neutrality goals.^[^ ^8^ ^]^ Recycling industrial wastes as substitutes for natural aggregates and ordinary Portland cement in concrete offers potential benefits by reducing carbon emissions and reducing the use of landfills.

To recycle and utilize these wastes, experts have conducted in‐depth research. Noor and colleagues incorporated coal bottom ash (CBA) as a fine aggregate into concrete and observed that as the percentage of CBA increased, the strength of the concrete decreased.^[^ ^9^ ^]^ Argiz and Menéndez introduced CBA as a gel material into cement and found that the concrete's resistance to chloride and sulfate corrosion improved with an increase in the CBA content (within the range of 25%).^[^ ^10^, ^11^ ^]^ Özkılıç et al.^[^ ^12^ ^]^ introduced varying proportions of waste glass aggregate (WGA) into a fixed ratio of alkali‐activated fly ash‐based geopolymer concrete. They conducted tests on its physical properties and observed that the concrete's performance was inversely related to the proportion of WGA but directly proportional to the NaOH concentration. They also established a strength model and derived an equation for predicting compressive strength. Karalar and Benzannache et al.^[^ ^13^, ^14^ ^]^ partially replaced cement with marble powder to produce reinforced concrete beams (RCB). They observed that as the proportion of marble powder increased, the type of cracks in RCB shifted from bending cracks to shear cracks. Additionally, the flexural load‐bearing capacity of the beams gradually decreased but remained within an acceptable range. Furthermore, the compressive strength of the concrete also exhibited a slight decrease. In order to investigate the impact of recycled Polyethylene Terephthalate (PET) on the physical properties and durability of high‐strength concrete, Qaidi et al.^[^ ^15^ ^]^ added it as fine aggregate into the concrete. They found that both the physical and mechanical properties as well as the durability of the concrete decreased. Therefore, it is recommended to use it only for non‐structural applications, and the replacement ratio should not exceed 25%. In order to enhance the bond performance of ribbed steel bars embedded in recycled aggregate concrete (RAC), Fayed et al.^[^ ^16^ ^]^ employed steel mesh fabric cylinders (SMF) of various diameters to confine the recycled concrete around the steel bars. They conducted center pull‐out tests on 12 mm diameter steel bars embedded in concrete with varying levels of recycled concrete aggregate (RCA) replacement. The results showed that SMF significantly improved the ultimate bond strength of the confined specimens. Additionally, they proposed a formula to calculate the ultimate bond strength of RAC.

Coal gangue is an industrial waste generated during coal mining and processing operations.^[^ ^17^ ^]^ In 2018, global coal production reached 7.85 billion tons, with coal gangue accounting for ≈117–157 million tons.^[^ ^18^ ^]^ Developing countries, particularly China, have accumulated ≈5 billion tons of coal gangue, leading to severe environmental pollution and high disposal costs.^[^ ^19^, ^20^, ^21^, ^22^ ^]^ Coal gangue contains significant amounts of siliceous and aluminous minerals, which give it a certain level of pozzolanic activity. Additionally, the chemical composition of coal gangue is similar to that of fly ash (FA), and research on the use of fly ash as a supplementary cementitious material has been conducted for nearly a century. Hence, research is underway on grinding coal gangue into powder and using it as a substitute for ordinary Portland cement.

Currently, research on coal gangue concrete primarily focuses on the use of coal gangue as recycled coarse aggregates and its incorporation as a gel material in concrete.^[^ ^22^, ^23^, ^24^, ^25^, ^26^, ^27^, ^28^, ^29^, ^30^ ^]^ Due to its lightweight and high‐strength characteristics, researchers have investigated the crushing of coal gangue for use as an aggregate for recycled concrete. Studies of its physical properties and failure mechanisms showed that substituting natural coarse aggregates with coal gangue in the production of C30 and C40 concrete was feasible. To investigate the static and dynamic mechanical properties of coal gangue aggregate concrete (CGAC) in mine support structures, Yao et al.^[^ ^31^ ^]^ conducted compressive strength tests, drop hammer impact tests, and split Hopkinson pressure bar (SHPB) tests. The comprehensive results indicated that CGAC could be used for supporting coal mining engineering projects, considering its favorable performance in terms of compressive strength and resistance to dynamic loading. Zhang et al.^[^ ^22^ ^]^ utilized the spontaneous combustion gangue aggregate (SCGA) contained in coal gangue as a substitute for natural aggregates. They investigated the influence of the SCGA replacement ratio and particle size distribution on the mechanical properties of concrete. Furthermore, they proposed a method for predicting the mechanical performance of coarse SCGA‐concrete. The results demonstrated that SCGA had a significant impact on the mechanical properties of concrete. Zhang et al.^[^ ^32^ ^]^ utilized coal gangue as an aggregate to prepare thermal insulation concrete. They found that when the replacement ratio of coal gangue reached 30%, the comprehensive strength of the concrete reached its optimum level. Zhang et al.^[^ ^33^ ^]^ replaced natural sand with coal gangue sand in concrete and observed that coal gangue sand weakened the adhesive properties in the transition zone between mortar and coarse aggregates.^[^ ^5^, ^34^, ^35^, ^36^, ^37^ ^]^ Researchers investigated various volcanic ash materials and added them to concrete; These materials could improve the mechanical properties of recycled aggregate concrete. Zhu et al.^[^ ^38^ ^]^ introduced coal gasification slag (CGS) as a gel material in concrete. After subjecting the concrete to 125 cycles of freeze‐thaw testing, CGS concrete exhibited strong frost resistance. Guan et al.^[^ ^39^ ^]^ investigated the degradation mechanism of coal gangue concrete under the combined effects of bending load and freeze‐thaw cycles. Su et al.^[^ ^40^ ^]^ found that adding thermally activated coal gangue powder as a gel material to concrete results in a denser interfacial transition zone between aggregates and the matrix. Wang et al.^[^ ^18^ ^]^ developed steam‐cured aerated concrete using coal gangue (CGC) and iron ore tailings. They analyzed the material composition of coal gangue, the calcination temperature, and the composition of hydration products using differential scanning calorimetry (DSC), thermogravimetric analysis (TGA), X‐ray diffraction (XRD), and scanning electron microscopy (SEM). The study revealed that coal gangue powder exhibited optimal volcanic ash activity when subjected to high‐temperature calcination at ≈600 °C, which could improve the microstructure of cement‐based systems. However, the high‐temperature calcination of coal gangue can release harmful substances, causing environmental pollution and posing health risks to humans.^[^ ^41^, ^42^ ^]^ Moreover, the calcination process consumes a significant amount of energy, leading to increased carbon emissions that contradict the original intention of achieving carbon peak and carbon neutrality. Therefore, in this study, CGP was directly used to replace Portland cement, investigating its impact on the performance of recycled aggregate concrete. There are currently relatively few papers in this field.

Sustainable concrete production necessitates the efficient use of energy and materials.^[^ ^43^, ^44^, ^45^, ^46^, ^47^ ^]^ To achieve this objective, this study was based on the research of experts and scholars worldwide.^[^ ^48^, ^49^, ^50^, ^51^ ^]^ It involved the utilization of coarse recycled aggregates obtained from construction and demolition waste (C&D) to replace natural aggregates at different substitution rates. Additionally, coal gangue micro‐scale powder is substituted for ordinary Portland cement (OPC) at various levels to produce recycled concrete. In this study, the influence of various factors on the strength of concrete was investigated by incorporating different percentages of CGP as a sole replacement, RA as a sole replacement, and varying the WCR. Furthermore, an orthogonal experiment was conducted to investigate the influence of different substitution percentages of RA (20%, 40%, and 60%), CGP substitution ratios of cement (10%, 15%, and 20%), and WCR values (0.45, 0.5, and 0.55) on the macroscopic mechanical properties of CGP‐RAC. Moreover, SEM, EDS, and XRD techniques were employed to perform microscopic investigations on the morphology of CGP, elemental composition, hydration products, and hydration mechanism to reveal the mechanism of the influence of CGP on the strength of RACs.

## 2 Materials and Specimen Preparations

### 2.1 Materials

**Figure 1:**
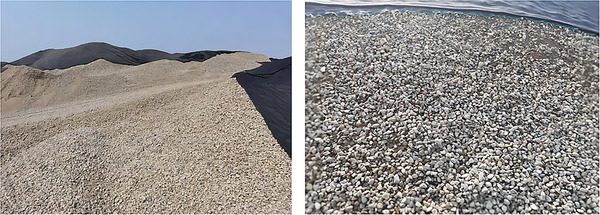
The production site of recycled aggregates.

**Figure 2:**
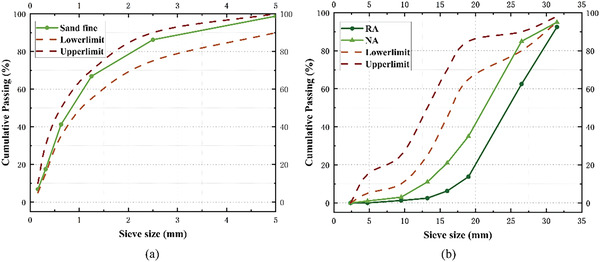
Grading curves of aggregates: (a) fine and (b) coarse aggregates.

In this study of CGP‐RAC, the main materials included RA, CGP, cement, natural sand, natural aggregates (NAs), and water. The recycled aggregates and natural aggregates used in this study were obtained from the Xijian Recycling Material Plant in Chengdu (**Figure** **1**). The particle size distribution, apparent density, bulk density, crushing index, water absorption, and moisture content of recycled aggregates and natural aggregates were measured in accordance with the current standards GB/T25177‐2010^[^ ^52^ ^]^ and JGJ52‐2006.^[^ ^53^ ^]^ The results showed that compared to natural crushed stone, the particle size distribution of recycled aggregates was significantly uneven. The bulk density of RA was ≈10% less than that of NA, which indicated that RA was lighter than NA. The crushing index of RAs was 1.6 times that of natural aggregates NAs, which indicated that RA had less strength compared to NA. The water absorption of RA was 3.4 times that of NA, and the moisture content of RA was 4.2 times that of natural aggregates. Overall, the various indicators were significantly weaker for RA than NA. The particle size distribution curves of RAs and NAs are shown in **Figure** **2**, and their physical properties are presented in **Table** **1**.

**Table 1:** Physical properties and chemical compositions of aggregates.

Test metrics	Apparent density [kg m^−3^]	Bulk density [kg m^−3^]	Crushing index [%]	Water absorption [%]	Moisture content [%]
NA	2664.67	1622.3	8.38	0.81	0.45
RA	1622.3	1453.43	13.66	2.78	1.9

**Figure 3:**
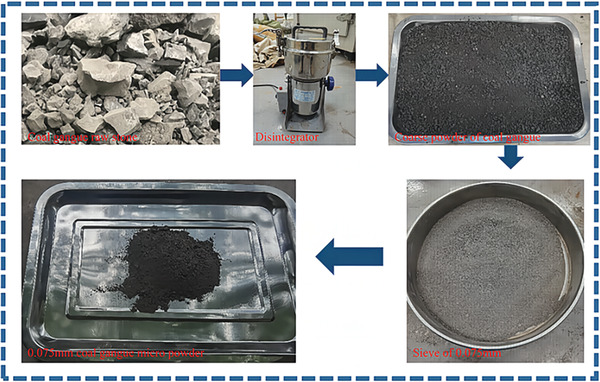
Preparation of CGP.

The natural raw coal gangue used in this study was sourced from Maping Mine in southwestern China. The XRD diffraction pattern revealed that the main components of the coal gangue were quartz, biotite, and pyroxen. The content of each component is presented in **Table** **2**. Large chunks of raw coal gangue were crushed, ground, and sieved through a mesh to obtain coal gangue micro‐scale powder with a diameter of less than 0.075 mm, as shown in **Figure** **3**.

**Table 2:** Oxides in coal gangue.

	SiO_2_	Al_2_O_3_	Fe_2_O_3_	CaO	K_2_O	MgO	TiO_2_	Others
Coal gangue	51.51%	17.98%	12.32%	7.62%	5.1%	2.68%	1.25%	1.54%

All the blended materials complied with the requirements of GB/T175, including ordinary Portland cement with a nominal strength of 42.5 MPa (P.O.42.5R), ordinary tap water, and continuously graded natural sand.^[^ ^54^ ^]^ The grading curve of the natural sand is shown in Figure 2.

### 2.2 Mix Proportion Design

Four concrete mix designs with a target strength of C30 were developed for this experiment. The compressive strength and splitting tensile strength of the four concrete mix designs were measured to investigate the influence of incorporating RA and CGP into concrete as well as the influence of WCR on the strength of RAC. The first group (**Table** **3**) was designed to investigate the effect of replacing cement with coal gangue powder (CGP) on concrete strength. The replacement rates of CGP for cement were 0% (control group), 10%, 20%, 30%, 40%, and 50%. Additionally, an equivalent set of experiments was designed with the same number of groups and dosage, where FA replaced cement as the control test. This was done to study the differences in the effects of CGP and conventional admixtures on concrete. The second group (**Table** **4**) was designed to investigate the impact of replacing NA with RA on concrete. The replacement ratios were 0%, 20%, 40%, 60%, 80%, and 100%. The third group (**Table** **5**) was designed to explore the influence of WCR on the mechanical properties of concrete. The designed WCR values were 0.45, 0.50, 0.55, and 0.60. The fourth group (**Table** **6**) was designed to investigate the influence of the simultaneous incorporation of CGP and RA on the strength of concrete. The use of an orthogonal table in an experimental design allowed for systematic testing, significantly reducing the number of required tests and shortening the development time. Building upon the results of the previous three groups of experiments, this study employed an orthogonal experimental design to investigate the effects of CGP and RA on the strength of concrete. By using an orthogonal table, the interactions and individual effects of CGP and RA on concrete strength were studied in a more efficient and structured manner. The factors investigated include the replacement ratio of CGP (A), the replacement ratio of RA (B), WCR (A), and their interactions. **Table** **7** presents the factors and levels after employing the L9 (3^3^) orthogonal column design. Thirty mixed proportions were established, resulting in a total of 276 cubic specimens for testing.

**Table 3:** Mix Proportions for CGP Single‐Factor Experiment (kg m^−3^).

Group	Cement	CGP	Water	NA	Sand
1 (10%)	262.5	29.17	175	1218.67	655.64
2 (20%)	233.34	58.33	175	1216.47	654.46
3 (30%)	204.17	87.5	175	1214.36	653.33
4 (40%)	175	116.67	175	1212.18	652.15
5 (50%)	145.83	145.84	175	1210	651
6 (0%)	291.67	0	175	1220.39	657.18

**Table 4:** Mix proportions for the RA single‐factor experiment (kg m^−3^).

Group	Cement	Water	Sand	NA	RA
1 (20%)	319.07	191.44	632.37	939.45	234.86
2 (40%)	344.61	206.77	610.93	680.7	453.8
3 (60%)	360.61	216.36	590.9	438.91	658.38
4 (80%)	390.84	234.5	572.18	212.51	850.03
5 (100%)	412.82	247.09	554.58	0	1029.86
6 (0%)	291.67	175	657.18	1220.39	0

**Table 5:** Mix proportions for the WCR experiment in RAC (kg m^−3^).

WCR	Water	Cement	Sand	NA	RA
0.45	216.36	480.8	590.9	438.91	658.38
0.5	216.36	432.72	590.9	438.91	658.38
0.55	216.36	393.38	590.9	438.91	658.38
0.6	216.36	360.61	590.9	438.91	658.38

**Table 6:** Mix proportions of the orthogonal experiment (kg m^−3^).

ID	combination	Cement	Sand	Water	RA	NA	CGP
1	A1B1C1	382.04	616.34	191.02	228.91	915.64	42.45
2	A1B2C3	415.96	579.76	220.22	645.96	430.64	73.41
3	A1B3C2	367.19	606.52	206.54	450.52	675.79	91.79
4	A2B1C3	396.98	584.07	220.54	650.65	433.76	44.11
5	A2B2C2	351.5	610.94	206.77	453.81	680.71	62.03
6	A2B3C1	306.54	638.04	191.59	236.97	947.87	76.64
7	A3B1C2	338.65	614.55	206.95	456.49	687.73	37.63
8	A3B2C1	296.21	641.05	191.67	238.08	952.34	52.27
9	A3B3C3	323.20	605.21	222.20	674.32	449.55	80.80

**Table 7:** Factor levels of the orthogonal experimental design.

Factor level	A	B	C
	WCR [%]	CGP replacement rate [%]	RA replacement rate [%]
1	0.45	10	20
2	0.5	15	40
3	0.55	20	60

### 2.3 Specimen Preparations

**Figure 4:**
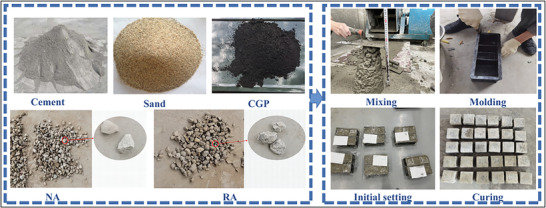
The process of concrete preparation.

Recycled aggregate has greater porosity and water absorption capacity than natural aggregate, which means that it can absorb some of the water in concrete, decreasing the water‐cement ratio of the concrete. To maintain the workability of the concrete, it is necessary to pre‐treat RA to reduce its water absorption rate before mixing.^[^ ^55^, ^56^ ^]^ The RA should be pre‐wetted by immersing it in water for 10 min and then draining it for 10 min to achieve a state of saturated surface dryness before incorporating it into the concrete mix. A 30 L capacity single‐axis forced mixer was used in this experiment (Yixuan HJW‐60, China). The gel materials, fine aggregates, and water were first placed into the mixer and stirred for 60–90 s, followed by the addition of coarse aggregates and continued mixing for 120 s. According to the requirements of GB/T50080,^[^ ^57^ ^]^ the slump test was conducted on the fresh concrete, and the slump of the recycled concrete was slightly higher than that of the ordinary concrete. The prepared concrete was poured into a plastic mold and placed on a vibrating table to compact the mixture until it became smooth and dense. After 24 h of curing at room temperature, the concrete was demolded. The specimens were cured at 100% humidity for 7 or 28 days. After natural drying, they were subjected to mechanical performance testing. The preparation process is shown in **Figure** **4**.

## 3 Experimental Methods

### 3.1 Compression and Splitting Tensile Tests

**Figure 5:**
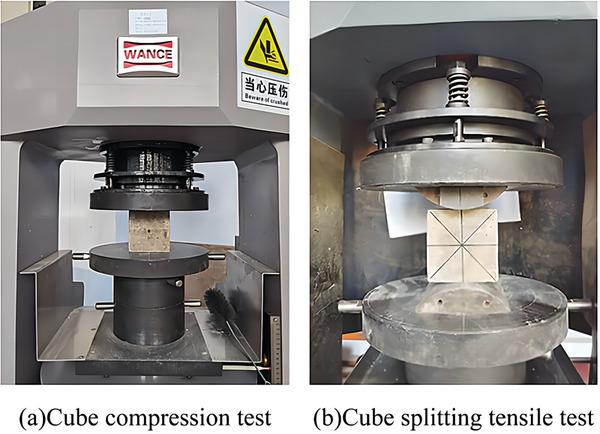
Compression and splitting tensile tests.

Compressive strength tests were conducted on concrete cubes after 3, 7, and 28 days of curing using a 3000 kN computer‐controlled hydraulic servo pressure testing machine (HCT206A, Wance). According to the requirements of GB/T50081,^[^ ^58^ ^]^ specimens with dimensions of 100 mm × 100 mm × 100 mm were used for the compressive strength test at a loading rate of 0.5 For the splitting tensile strength test, a loading rate of 0.05 MPa s^−1^ was applied. The steps for the splitting tensile strength test are as follows: 1) The specimen was removed from the curing water tank and checked for any damage, honeycombs, or surface defects. Any problematic specimens were discarded. 2) The axial centerline was marked on the surface of the specimen to determine the location of the splitting plane. 3) The flatness and height differences of the upper and lower pressure plates of the testing machine were checked to ensure compliance with the specifications. 4) A steel pad was placed in the center position of the pressure plate of the testing machine, and the specimen was positioned at the center of the steel pad. 5) The parameters of the testing machine were set; the loading rate was set to 0.05 MPa s^−1^. 6) The testing machine was started to conduct the test, the specimen was observed under tension, and the data were recorded. To reduce variability, the test results for three samples of each mix proportion were averaged. Photographs of the compressive strength test and split tensile strength test are shown in **Figure** **5**. The strength calculation formulas are given by Equations (1) and (2).

(1) $$f_{\mathrm{cu}}=\frac{F}{A}$$

(2) $$f_{t}=\frac{2F}{\pi F}=\frac{F}{A}$$

where *f*_cu_ and *f*_t_ represent the compressive strength and split tensile strength of the concrete specimens, respectively, (MPa); *F* represents the peak load at specimen failure, (N); and *A* is the cross‐sectional area of the specimen subjected to compressive or tensile stress, (mm^2^).

### 3.2 ANOVA

ANOVA is the statistical treatment most commonly applied to experimental results. It can be used to divide the influence of the factors from those of experimental error. The *F* value indicates the ratio of the mean square (MS) of each factor to that of the experimental error and is analyzed by the *F* test.^[^ ^56^ ^]^ The sum of squared deviations (SS) reflects the differences in experimental results caused by changes at every level of each factor or error and is calculated as follows:

(3) $$SS=\sum_{j=1}^{m}{\left(y_{j}-\bar{y}\right)}^{2}$$

where *y*_j_ is the value of the result of each trial, and $\bar{y}$ is the arithmetic average of *y*_j_.

The MS of each factor or experimental error is SS divided by the number of degrees of freedom (DF) and obtained by Equation (4).

(4) MS=SS/DF

The critical value of the *F* value (*F*_α_) for a different level of significance, such as α = 0.01; 0.05, can be found in the *F*‐statistic distribution table. When *F* > *F*_0.01_, the factor effect for the results is highly significant to the index and marked as “***”. If *F*_0.05_ < *F* ≤ *F*_0.01_, then the corresponding factor has a significant effect on the index and is marked as “**”. If *F*_0.1_ < *F* ≤ *F*_0.05_, then the corresponding factor has a minimal impact on the index and is marked as “*”. If *F* < *F*_0.1_, then the corresponding factor has no impact on the index and is marked as “–”.^[^ ^59^ ^]^

### 3.3 Microstructure Analysis

**Figure 6:**
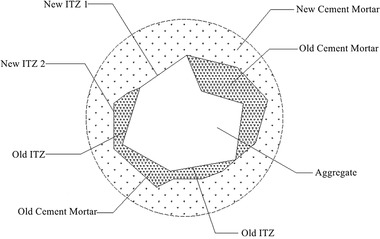
Microstructure of recycled concrete.

**Figure 7:**
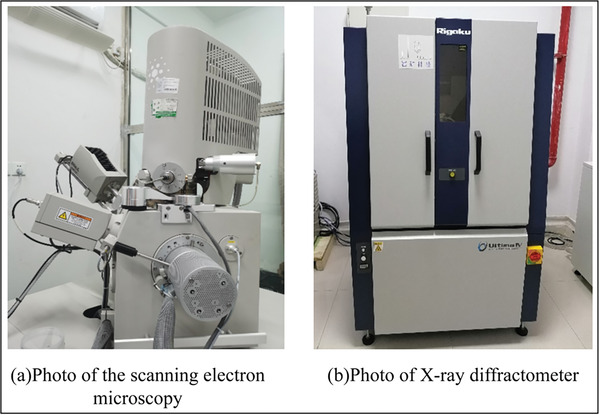
Photographs of instruments used for SEM and XRD.

The recycled concrete consists of a “five‐phase” structure, which includes coarse aggregates, old interfacial transition zone, old mortar, new interfacial transition zone, and new mortar, as illustrated in the diagram (**Figure** **6**). This structure results in the formation of two types of new interfaces (old mortar to new mortar and old aggregates to new mortar). The interfacial transition zone is often weak in concrete, and internal damage in concrete typically initiates at this region. In addition, the strength of hydration reactions is a crucial factor influencing concrete strength. Therefore, the performance of hydration reactions after partially replacing cement with CGP determines the performance of CGP‐RAC. Microstructural analysis was performed to observe the new interfacial transition zone, assess the degree of hydration reactions, and understand the microstructure of CGP‐RAC. The microstructure of CGP‐RAC was examined and magnified using SEM (FEI Inspect, Thermo Fisher). Elemental species, concentration ratios, and molar ratios were identified and determined using an EDS analyzer (NCA X‐Act, OBR). Hydration products were analyzed using an XRD instrument (ULTIMA IV, Rigaku) (**Figure** **7**).

## 4 Experimental Results

### 4.1 Analysis of Single‐factor Experiments

#### 4.1.1 Substitution rate of CGP

**Figure 8:**
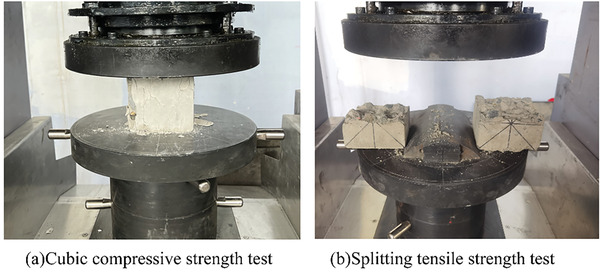
Axial Compression and Split Tensile Tests.

**Figure 9:**
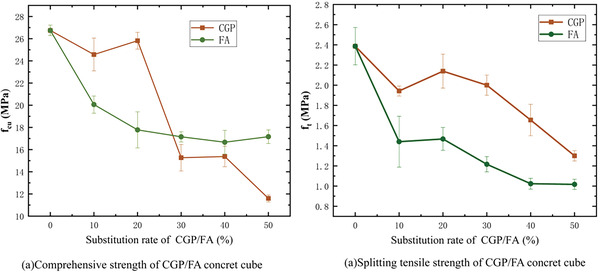
*f*_cu_ and *f*_t_ of concrete with different substitution rates of CGP and FA.

**Figure** **8** displays photos of specimens after axial compression and split tensile strength tests. Detailed information regarding the 28‐day strength of concrete with CGP and fly ash (FA) addition is provided in **Figure** **9**.

In Figure 9, the splitting tensile strength and compressive strength exhibited similar trends for concrete with CGP and FA. Therefore, when the substitution of CGP was less than 20%, the loss in compressive strength of concrete was ≈3–7%, which was considered reasonable.

#### 4.1.2 Substitution Ratio of RA

**Figure 10:**
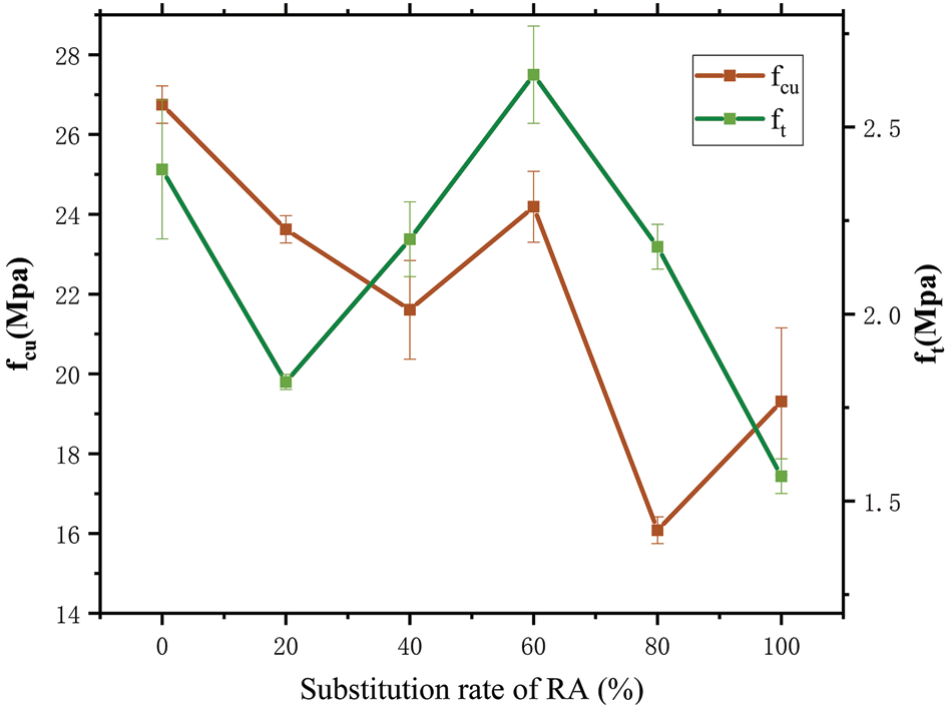
*f*_cu_ and *f*_t_ of concrete with different replacement rates of RA.

Detailed information on the 28‐day compressive strength and split tensile strength of concrete with different substitution ratios of RA is provided in **Figure** **10**. The concrete was strongest when the replacement ratio of recycled aggregate was ≈50–60%. The results were consistent with the findings of previous researchers in the field.^[^ ^60^, ^61^, ^62^, ^63^ ^]^ This was attributed to the rough surface and high porosity of the recycled aggregates, which resulted in a water absorption rate three times greater than that of natural aggregates. During the concrete preparation process, the recycled aggregates tended to absorb a significant amount of water (considered in the mix design). The additional water absorbed and stored by the recycled aggregates was gradually released with increasing curing time. The released water could effectively reduce the heat generated by cement hydration, thereby balancing the temperature difference between the interior and exterior of the concrete. This process improved the mechanical properties of the concrete and is known as the “internal curing” effect. The internal curing effect could be better achieved when sufficient recycled aggregate was present, which allowed for adequate absorption and release of water. The presence of an excessive amount of recycled aggregate could have drawbacks such as less inherent strength and poor gradation, which could lead to a reduction in the overall strength of the concrete. This could explain why the concrete was strongest when the replacement ratio of recycled aggregate was ≈50–60%.

#### 4.1.3 WCR

**Figure 11:**
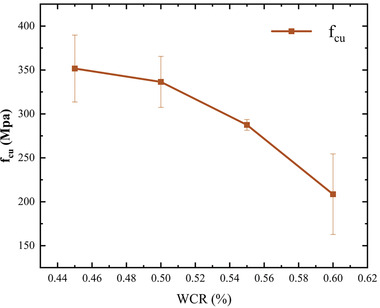
*f*_cu_ values of concrete with different WCRs.

**Figure** **11** presents the compressive strength of the concrete cubes at 28 days for different WCRs. The compressive strength of recycled concrete decreased with increasing WCR. However, increasing the WCR was beneficial for improving the workability of the concrete, leading to increased slump and enhanced workability performance. Therefore, when designing orthogonal experiments, both concrete strength and workability should be considered. WCRs of 0.45, 0.5, and 0.55 were selected as the levels for the A.

### 4.2 Analysis of Orthogonal Experiment

#### 4.2.1 Orthogonal Analysis

**Figure 12:**
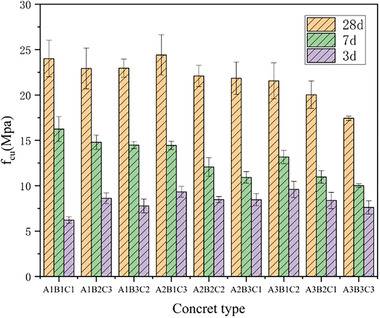
Effects of RAC replacement rate, CGP content, and water‐cement ratio on *f*_cu_ of CGP‐RAC.

**Figure 13:**
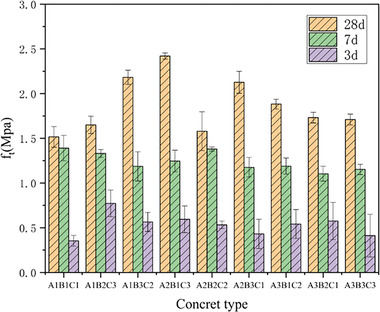
Effects of RAC replacement rate, CGP content, and water‐cement ratio on f_t_ of CGP‐RAC.

Detailed information regarding *f*_cu_ at 3, 7, and 28 days from the orthogonal experiment can be found in **Table** **8** and **Figure** **12**. The control group consisted of concrete samples with 60% and 0% replacement of RA. When the curing age was 3 days, the *f*_cu_ values of all specimens in the orthogonal experiment were more than 32% of the 28‐d *f*_cu_ values; Specimen Z7 reached 44.5%. For the control group, the values of the strength at 3 days were 33.3% and 31.9% of the values of the 28‐day strength. When the curing age was 7 days, every specimen had developed compressive strength that exceeded 50% of the 28‐day compressive strength. In particular, specimen Z1 reached a high compressive strength of 65.1%. The 7‐day compressive strengths of the reference group were 64.3% and 63.5% of the corresponding 28‐day strengths. At a curing age of 28 days, the specimens achieved a maximum compressive strength of 25.42 MPa, which was 75.6% and 73.6% of the compressive strengths of the reference group specimens. The development of ft for all specimens in the orthogonal experiment was similar to that of *f*_cu_. It showed a relatively fast initial development rate but ultimately exhibited varying degrees of decrease compared to the reference group (**Table** **9** and **Figure** **13**). The maximum ft value was 2.43 MPa, which corresponded to 85.6% and 77.4% of the reference group. The fast rate of strength development in CGP‐RAC could be attributed to the high porosity of the reinforced concrete, which allowed it to absorb more surface water, increase the contact area with the cementitious materials, and enhance the hydration reaction rate. The strength of CGP‐RAC developed rapidly during the early curing period. However, due to the presence of CGP and RA, its strength was still less than that of conventional concrete.

**Table 8:** *f*_cu_ of CGP‐RAC from the orthogonal test.

ID	WCR [%]	CGP [%]	RA [%]	*f*_cu_/MPa		
	A	B	C	3d	7d	28d
A1B1C1	0.45	10	20	8.22	16.24	24.95
A1B2C3	0.45	15	60	8.63	14.8	23.88
A1B3C2	0.45	20	40	8.13	14.48	24.83
A2B1C3	0.5	10	60	9.32	14.45	25.42
A2B2C2	0.5	15	40	8.47	12.07	22.1
A2B3C1	0.5	20	20	8.46	10.93	21.85
A3B1C2	0.55	10	40	9.6	13.18	21.57
A3B2C1	0.55	15	20	8.38	10.91	20.91
A3B3C3	0.55	20	60	7.62	10.01	17.43
Control group1	0.5	0	60	11.2	21.62	33.64
Control group2	0.5	0	0	11.04	21.93	34.52

**Table 9:** *f*_t_ of CGP‐RAC from the orthogonal test.

ID	WCR [%]	CGP [%]	RA [%]	*f*_t_/MPa		
	A	B	C	3d	7d	28d
A1B1C1	0.45	10	20	0.45	1.39	1.79
A1B2C3	0.45	15	60	0.77	1.33	1.78
A1B3C2	0.45	20	40	0.57	1.19	2.43
A2B1C3	0.5	10	60	0.59	1.25	2.43
A2B2C2	0.5	15	40	0.53	1.38	1.58
A2B3C1	0.5	20	20	0.53	1.18	2.13
A3B1C2	0.55	10	40	0.54	1.2	1.88
A3B2C1	0.55	15	20	0.69	1.11	1.74
A3B3C3	0.55	20	60	0.54	1.14	1.47
Control group1	0.5	0	60	0.65	1.59	2.84
Control group2	0.5	0	0	0.57	2.02	3.14

#### 4.2.2 Range and Trend Chart

**Figure 14:**
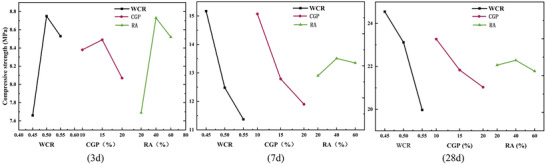
Effects of different factors on the compressive strength of CGP‐RAC.

**Figure 15:**
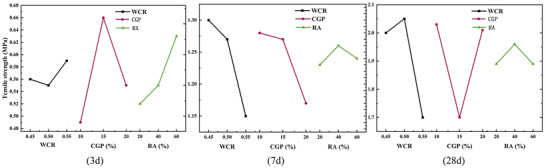
Effects of different factors on the tensile strength of CGP‐RAC.

Range, trend, and variance analyses were conducted to evaluate the strength of CGP‐RAC for different CGP replacement ratios, RA replacement ratios, and water‐cement ratios. The results are presented in **Tables** **10** and **11**. By comparing the ranges of various factors, the influences of different factors on the *f*_cu_ of CGP‐RAC at a curing age of 3 days could be ranked as follows: A > C > B. As the WCR increased, the *f*_cu_ of CGP‐RAC increased. This was because a higher water density resulted in a larger contact area of the gel material, leading to faster hydration and strength development. However, when considering the effects on ft at a curing age of 3 days, the ranking was B > C > A, which was opposite to the results for *f*_cu_. The specific reasons for this discrepancy needed further analysis. At curing ages of 7 and 28 days, the ranking could be determined as A > B > C. After the curing age reached 7 days, the amount of CGP became the second influencing factor, and more CGP led to lower strength. According to **Figures** **14**, **15**, the mid‐term and later‐stage compressive strength and split tensile strength of CGP‐RAC decreased with increasing the water‐cement ratio, which was consistent with ordinary concrete. The mid‐term and later‐stage compressive strength as well as the split tensile strength of CGP‐RAC decreased with increasing replacement rate of CGP. A replacement rate of 40% for RA was the optimal replacement rate, which differed from the results of the single‐factor experiment in Experiment 2. Further analysis using variance analysis was required to determine whether this difference was due to experimental errors.

**Table 10:** Range analysis of *f*_t_.

Index factor	3d			7d			28d		
	A	B	C	A	B	C	A	B	C
K_1_	1.69	1.48	1.57	3.91	3.84	3.68	6	6.1	5.66
K_2_	1.65	1.99	1.64	3.81	3.82	3.77	6.14	5.1	5.89
K_3_	1.77	1.64	1.9	3.45	3.51	3.72	5.09	6.03	5.68
k_1_	0.56	0.49	0.52	1.3	1.28	1.23	2	2.03	1.89
k2	0.55	0.66	0.55	1.27	1.27	1.26	2.05	1.7	1.96
k3	0.59	0.55	0.63	1.15	1.17	1.24	1.7	2.01	1.89
R	0.04	0.17	0.11	1.15	0.11	0.03	0.35	0.33	0.07
Order	B>C>A			A>B>C			A>B>C		

**Table 11:** Range analysis of *f*_cu_.

Index factor	3d			7d			28d		
	A	B	C	A	B	C	A	B	C
K_1_	22.98	25.14	23.06	45.52	43.87	38.07	73.66	71.94	67.71
K_2_	26.25	25.48	26.2	37.44	37.78	39.73	69.37	66.89	68.5
K_3_	25.6	24.21	25.57	34.1	35.41	39.26	59.91	64.11	66.73
k_1_	7.66	8.38	7.69	15.17	14.62	12.69	24.55	23.98	22.57
k2	8.75	8.49	8.73	12.48	12.59	13.24	23.12	22.3	22.83
k3	8.53	8.07	8.52	11.37	11.8	13.09	19.97	21.37	22.24
R	1.09	0.42	1.04	3.8	2.82	0.55	4.58	2.61	0.59
Order	A>C>B			A>B>C			A>B>C		

#### 4.2.3 Analysis of Variance

From **Tables** **12** and **13**, the factors A, B, and C had no significant impact on the 3‐day compressive strength of CGP‐RAC. This may be attributed to the fact that the cement hydration reaction has not fully developed at this early stage. Therefore, when comparing and selecting the optimal mix proportions, early strength indicators should not be the sole determining factor. According to the results in Table 11, factors A and B significantly influenced the 7‐day and 28‐day *f*_cu_ values of CGP‐RAC, while the effect of factor C was relatively small. Therefore, when comparing and selecting the optimal mix proportions, it was important to focus on the mid‐term and later strength indicators. In the single‐factor analysis, the replacement rate of RA had a significant impact on the strength of CGP‐RAC. However, it may not have shown significance in the variance analysis. Therefore, the selection of the replacement rate of RA (C) should follow the results of the single‐factor analysis.

**Table 12:** ANOVA table of *f*_cu_.

Factor	Significance criterion	DF	3d			7d			28d		
			SS	*F* value	Significance	SS	*F*‐value	Significance	SS	*F*‐value	Significance
WCR(A)	*F*_0.1_(2,6) = 3.46 *F*_0.05_(2,6) = 5.14 *F*_0.01_(2,6) = 10.925	2	1.997	1.67	–	22.984	176.8	***	32.995	13.2	***
CGP%(B)		2	0.288	0.24	–	12.697	97.68	***	10.504	4.2	*
RA%(C)		2	1.84	1.54	–	0.488	3.75	*	0.5242	0.21	–
Error		6	3.59			0.3922			7.497		

**Table 13:** ANOVA table of *f*_t_.

Factor	Significance criterion	DF	3d			7d			28d		
			SS	*F* value	Significance	SS	*F*‐value	Significance	SS	*F*‐value	Significance
WCR(A)	*F*_0.1_(2,6) = 3.46 *F*_0.05_(2,6) = 5.14 *F*_0.01_(2,6) = 10.925	2	0.0024	0.179	*	0.039	5.42	**	0.217	1.26	–
CGP%(B)		2	0.045	3.39	–	0.023	3.17	–	0.208	1.21	–
RA%(C)		2	0.02	1.49	–	0.0014	0.19	–	0.011	0.063	–
Error		6	0.04			0.022			0.517		

According to Tables 12 and 13, the three factors, WCR (A), CGP replacement rate (B), and RA replacement rate (C), did not have a significant influence on the split tensile strength of CGP‐RAC at 3, 7, and 28 d. Based on the results in Table 9, it was evident that only the CGP replacement rate (B) had a certain impact on the split tensile strength at 3 d. Additionally, the WCR (A) showed a significant influence on the split tensile strength at 7 d. The reason for this could be attributed to the small differences in the levels of the factors chosen in the orthogonal experiment and the relatively small variation in the split tensile strength of CGP‐RAC, with a maximum variation of 0.64 MPa. As a result, the sensitivity of the split tensile strength of CGP‐RAC to the variations in the levels of the three factors was small, which masked the significance of these factors. Therefore, conducting multiple comparisons between the levels had limited significance in this case. It would be more meaningful to rely on single‐factor analysis to assess the effects of each factor independently.

Based on the comprehensive analysis above, we concluded that the water‐cement ratio (Factor A) at the level of 0.45 (A1) was optimal in the range analysis trend graphs (Figure 13 for 7 d and 28 d and Figure 14 for 7 d). Based on the variance analysis, Factor A, which primarily considered the mid‐term and late‐term strength indicators of CGP‐RAC, suggested that the optimal level for Factor A was 0.45 (A1). The extreme difference analysis and variance analysis results for Factor B, the CGP replacement ratio, were consistent with those for Factor A. Therefore, the optimal level for Factor B was 10% (B1). Based on the results of the single‐factor analysis, the optimal level for Factor C, the RA replacement ratio, should be 60% (C3). Based on the analysis, the optimal combination of factors was A1B1C3. However, this specific combination was not included in the orthogonal experiment. The closest option available was the 4th test group, which corresponded to A2B1C3. The concrete mix proportion and the corresponding strength values are presented in **Table** **14**.

**Table 14:** Strength of the best mix ratio.

	WCR [%]	CGP [%]	RA [%]	*f*_cu_/MPa			*f*_t_/MPa		
				3d	7d	28d	3d	7d	28d
CGP‐RAC	0.5	10	60	9.32	14.45	25.42	0.59	1.25	2.43

### 4.3 Microanalysis Results

In the study conducted in Section 3, we found that the WCR and the replacement rate of CGP were the main factors influencing the performance of CGP‐RAC. To investigate the effects of CGP and WCR on cement hydration, concrete specimens from A2B1C3 (referred to as Z4) and A3B3C3 (referred to as Z9) combinations were selected, and their cross sections were monitored and analyzed. Z4 had a WCR of 0.5 and a CGP replacement rate of 10%, while Z9 had a WCR of 0.55 and a CGP replacement rate of 20%. Both Z4 and Z9 had an RA replacement rate of 60%. They had the greatest and weakest strengths, respectively, making them suitable for investigating the mechanisms by which WCR and CGP affect the strength of CGP‐RAC.

#### 4.3.1 SEM Analysis

**Figure 16:**
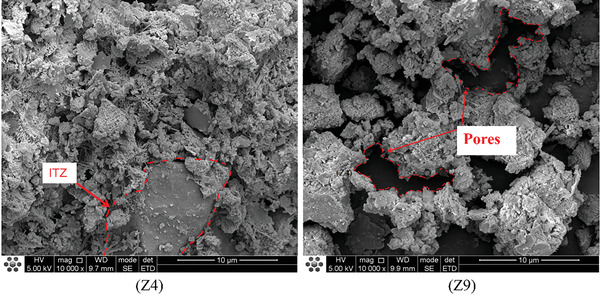
SEM micrographs of CGP‐RAC (10000 times magnification).

**Figure 17:**
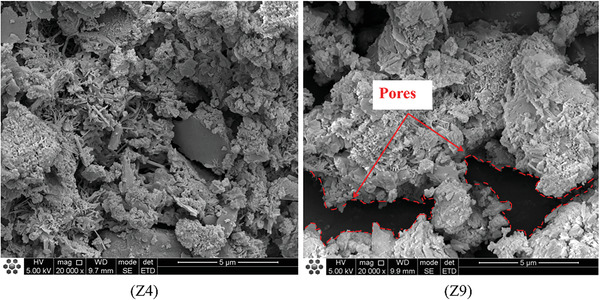
SEM micrographs of CGP‐RAC (20000 times magnification).

**Figure 18:**
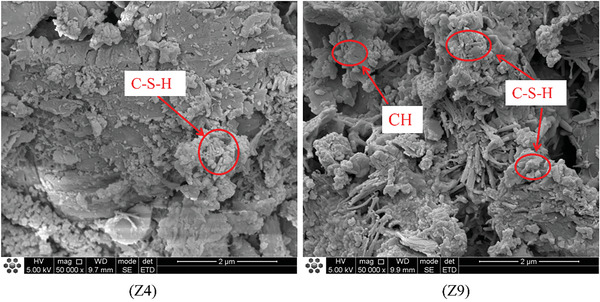
SEM micrographs of CGP‐RAC (50000 times magnification).

**Figures** **16**, **17**, **18** show SEM images of Z4 and Z9. From the analysis of the microstructure, both concrete mixtures exhibited a certain layered structure on the surface, primarily consisting of C‐H‐S gel and calcium hydroxide (CH), as well as needle‐shaped crystalline ettringite (AFt). The C‐H‐S gel and calcium hydroxide (CH) effectively filled the pores inside the recycled concrete, thereby enhancing the compactness of the cement matrix. In the SEM images at a magnification of 1 00 000 times, the surface of Z4 concrete exhibited a denser distribution of C‐H‐S gel and calcium hydroxide (CH). From the SEM images at magnifications of 20 000 times and 10 000 times, the surface of Z9 concrete contained some micro‐pores with a width exceeding 10 micrometers. These micro‐pores were the main cause of crack formation, extensive water absorption, and insufficient strength in the concrete. However, these micro‐pores significantly reduced the density of the interfacial transition zone in the concrete. Therefore, the internal structure of Z4 concrete was more compact with fewer voids and cracks and a higher density in the interfacial transition zone. This also explained the experimental result of a 30% decrease in compressive strength for Z9 concrete compared to Z4. The lower density and quantity of C‐H‐S gel and calcium hydroxide (CH) in Z9 may have been attributed to the influence of coal gangue micro‐scale powder on the cement hydration process. The greater the substitution rate of coal gangue micro‐scale powder, the greater the impact on hydration, resulting in a lower density of hydration products such as C‐H‐S and CH.

#### 4.3.2 Analysis of EDS

**Figure 19:**
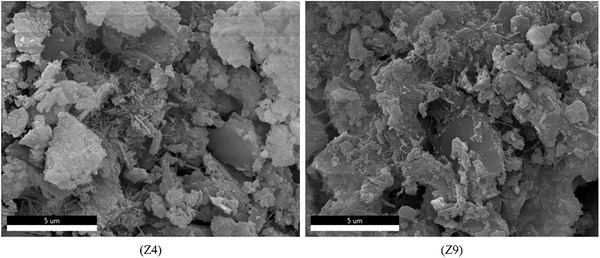
Scanning areas of EDS.

**Figure 20:**
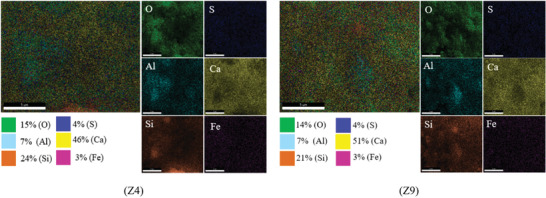
Chemical element distributions.

**Figure 21:**
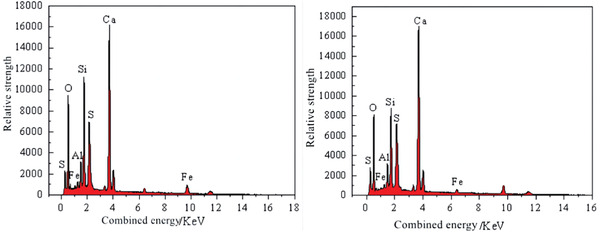
EDS energy spectra.

The analysis of EDS data can be divided into qualitative analysis and quantitative analysis. Qualitative analysis in EDS allows for rapid identification of the elements present in a test sample. Quantitative analysis, in contrast, builds upon qualitative analysis by calculating the relative intensities of signals to determine the concentration ratios or molar ratios of the corresponding elements. The chemical element distribution and energy spectrum of the EDS scans for the Z4 and Z9 test groups are shown in **Figures** **19**, **20**, **21**. The results of the quantitative analysis are shown in **Tables** **15**and **16**.

**Table 15:** Quantitative results of the analysis of EDS for group Z4.

Element	Apparent concentration [%]	wt.%	Atomic percentage [%]
O	15	43.3	62.9
Al	7	2.8	2.4
Si	24	10.4	8.6
S	4	8.8	6.4
Ca	46	32.5	18.8
Fe	3	2.1	0.9

**Table 16:** Quantitative results of the analysis of EDS for group Z9.

Element	Apparent concentration [%]	wt.%	Atomic percentage [%]
O	14	41.6	61.5
Al	7	3	2.6
Si	21	8.8	7.4
S	4	9	6.7
Ca	51	35.7	21
Fe	3	1.9	0.8

Figures 20 and 21 show that the CGP‐RAC was primarily composed of O, Ca, Si, S, and Al. Comparing the Z9 and Z4 groups, the major difference was in the percentage of Ca ions, which increased from 46% to 51%, and the percentage of Si ions, which decreased from 24% to 21%. The compositions of other elements remained relatively consistent between the two groups.

The calcium‐to‐silicon ratio (Ca/Si) in cement was an important factor that influenced the strength of the cementitious bond. Silicate cement is composed of minerals such as C3S, C2S, C3A, and others. These minerals undergo hydration reactions to form a gel‐like substance called calcium silicate hydrate (C‐S‐H), which significantly affects the strength and durability of the cement. Zhu et al.^[^ ^64^ ^]^ found that in low Ca/Si ratio cement, the C‐S‐H structure was more stable and ordered, with smaller cracks. In high Ca/Si ratio cement, the C‐S‐H structure was more disordered, and larger cracks were observed. According to Tables 15 and 16, the molar ratio of calcium to silicon (Ca/Si) in the CGP‐RAC specimens of the Z4 group was 2.18, while the Ca/Si molar ratio in the Z9 group was 2.84. In comparison to the Z4 group, the Z9 group showed an increase of 30% in the calcium‐to‐silicon ratio. However, the differences in the molar ratios of other elements were within 4% in both groups. Based on this, it could be inferred that the difference in strength between the CGP‐RAC samples could be attributed to the variation in the calcium‐to‐silicon ratio. Furthermore, the CGP‐RAC samples with a lower calcium‐to‐silicon ratio exhibited higher strength than those with a higher calcium‐to‐silicon ratio at 3, 7, and 28 days.

The higher calcium‐to‐silicon ratio in the Z9 group compared to the Z4 group was attributed to the adsorption effect of FA on alkaline substances. In cementitious materials, Ca(OH)_2_ exhibits alkaline properties, while SiO_2_ exhibits acidic properties. When fly ash is present, it selectively adsorbs Ca^2+^ from the cementitious matrix while not adsorbing Si^4+^. Therefore, the presence of FA in cementitious materials can result in the presence of free calcium ions, leading to an increase in the Ca/Si ratio of the material. CGP, being a volcanic ash‐like material with similar physical and chemical properties to FA, also exhibits the same characteristic of adsorbing calcium ions. The higher Ca/Si ratio in the Z9 group compared to the Z4 group could be attributed to the higher content of coal gangue powder in the Z9 group. As the dosage of coal gangue powder increased, more free Ca^2+^ ions were present in the cement matrix, resulting in a larger Ca/Si ratio.

#### 4.3.3 Analysis of the Hydration Mechanism

**Figure 22:**
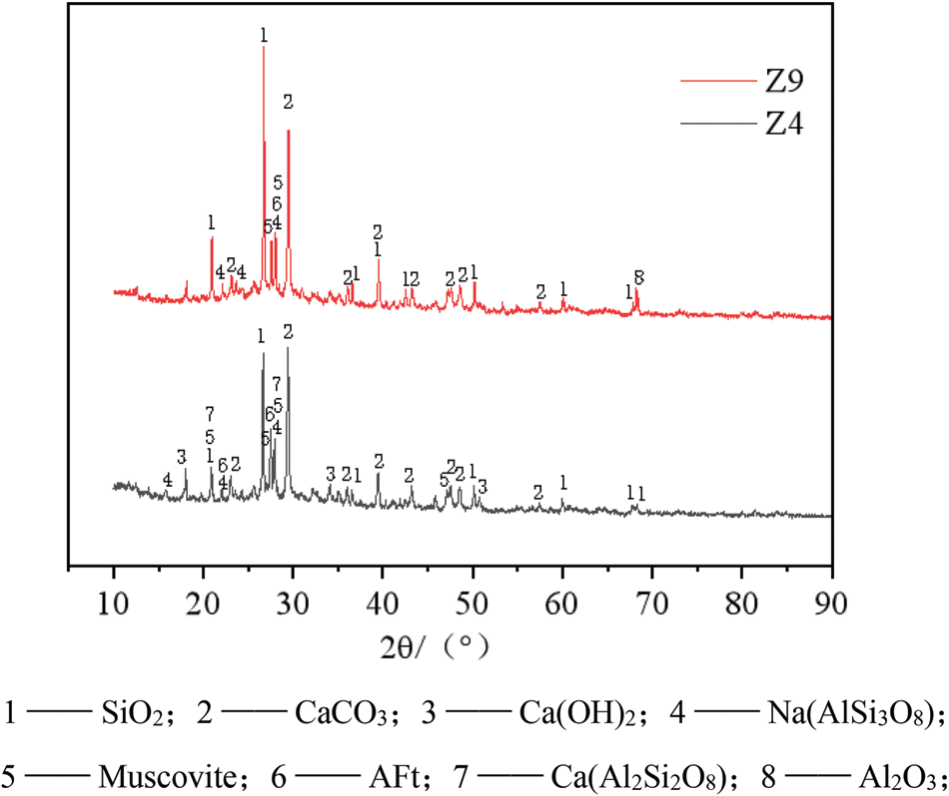
XRD spectra of the hydration products of coal gangue‐recycled concrete.

To further investigate the influence of CGP on the hydration reaction mechanism, an XRD analysis was performed on the Z4 and Z9 test groups to analyze the hydration products. The test results are shown in **Figure** **22**.

**Figure 23:**
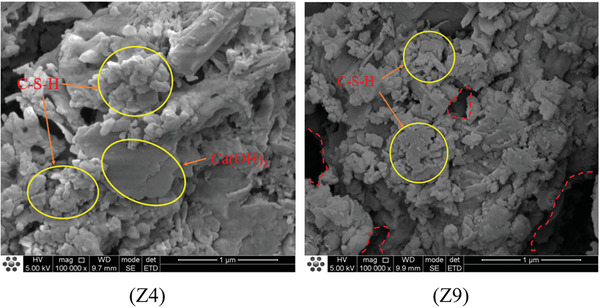
Micromorphology of coal gangue – recycled aggregate concrete (1 00 000×).

In Figure 22, at diffraction angles of 20.9°2θ and 26.6°2θ, the diffraction peaks of SiO_2_ were more pronounced in both the Z4 and Z9 groups. However, the SiO_2_ diffraction peak was more intense for Z9 than Z4, indicating the presence of a certain amount of SiO_2_ crystals in the composite paste of both groups, with a higher concentration in Z9. Cement mainly consists of dicalcium silicate (C2S) and tricalcium silicate (C3S). When cement reacts with water, it undergoes hydration to form calcium silicate hydrate (C‐S‐H). Since C‐S‐H does not have a crystalline structure, it is difficult to determine its presence through XRD analysis. However, in the SEM micrograph at a magnification of 1 00 000x, we clearly observed the cloud‐like morphology of the C‐S‐H gel, as shown in **Figure** **23**. Therefore, it was confirmed that the hydration reaction occurred within the paste, and the presence of SiO_2_ in the paste indicated the residue after hydration. At this stage, CaO has been completely consumed in the hydration reaction. Due to the relatively incomplete hydration reaction in the Z9 group, there was more remaining SiO_2_ in the paste, resulting in greater intensity of the SiO_2_ diffraction peak.

Coal gangue is a volcanic ash‐like supplementary cementitious material that contains a significant amount of amorphous SiO_2_ and Al_2_O_3_. It undergoes secondary hydration reactions with components in cement, such as Ca(OH)_2_, resulting in the formation of low‐alkalinity hydrated calcium silicate (C‐S‐H) gel. This phenomenon is commonly referred to as the volcanic ash effect. Figure 22 shows that the XRD spectrum of the Z4 group coal gangue‐recycled concrete exhibited diffraction peaks of Ca(OH)_2_ at 18.1°2θ, 34°2θ, and 50.8°2θ. Additionally, in Figure 23a, layered Ca(OH)_2_ could be identified. In contrast, Ca(OH)_2_ could not be observed in the XRD or SEM images of the Z9 group. This was attributed to the higher dosage of 10% CGP in Z9, which led to more consumption of Ca(OH)_2_ due to secondary hydration reactions. Furthermore, the water‐to‐binder ratio of Z9 was 0.05 greater than that of Z4. The increase in water content could result in the leaching and dissolution of Ca(OH)_2_, leading to the loosening of the cementitious matrix structure in Z9. Therefore, Ca(OH)_2_ could not be observed in Z9. This explained why, despite the more intense secondary hydration reaction in the internal structure of Z9 coal gangue recycled concrete compared to Z4, its compressive strength decreased by 30%. However, even the maximum strength achieved by Z4 reached only 73.6% of the compressive strength and 77.4% of the splitting tensile strength of ordinary concrete. This indicated that due to the differences in composition, the use of coal gangue micro‐scale powder as a replacement for cement severely hindered the original hydration reaction, resulting in poor development of hydration products and a decrease in concrete strength. While CGP exhibited volcanic ash activity and underwent secondary hydration reactions in the cementitious matrix, resulting in the formation of a small amount of hydration products with a certain strength contribution, it was not sufficient to compensate for the strength loss caused by the hindered primary hydration reaction.

## 5 Conclusion

The purpose of this study was to investigate the comprehensive utilization of coal gangue and construction solid waste. Coal gangue powder was used to partially replace cement, and construction solid waste was used as coarse aggregate to prepare concrete. By conducting single‐factor experiments, orthogonal experiments, and microscopic performance tests, combined with theoretical analysis, the optimal mix proportion of CGP‐RAC, factors affecting the mechanical properties of CGP‐RAC, the strength development behavior of CGP‐RAC, and the mechanism of the influence of CGP‐RAC were obtained. The main conclusions were as follows:

1) a) The influence of various factors on the early strength of CGP‐RAC was not significant. However, the compressive strength and splitting tensile strength of CGP‐RAC decreased with increasing water‐cement ratio. Therefore, the water‐cement ratio should be controlled between 0.45 and 0.5. The compressive strength and splitting tensile strength of CGP‐RAC decreased with increasing replacement rate of CGP‐RAC. The replacement rate of coal gangue micro‐powder should be controlled within 20%. The compressive strength and splitting tensile strength of CGP‐RAC exhibited wave‐like variations with increasing replacement rate of RA. Initially, they decreased, then increased, and finally decreased again. The optimal replacement rate of RA was 60%.
  b) The orthogonal experimental design resulted in the following mixture proportions for CGP‐RAC: WCR of 0.5, CGP replacement rate of 10%, and RA replacement rate of 60%. The mixture ratio was as follows: cement:sand:water:recycled aggregate:natural aggregate:coal gangue powder = 1:1.59:0.55:1.64:1.09:0.11. For this mixture proportion, the CGP‐RAC exhibited compressive strengths of 9.32, 14.45, and 25.42 MPa at 3, 7, and 28 days, respectively. The split tensile strengths were 0.59, 1.25, and 2.43 MPa at 3, 7, and 28 days, respectively. The utilization of coal gangue and recycled aggregates as solid waste was maximized while ensuring that the CGP‐RAC met the basic strength requirements.
2) Microscopic effects of coal gangue on the mechanical properties of CGP‐RAC.
  a) As the replacement ratio of CGP increased, the internal structure of CGP‐RAC became more porous and looser.
  b) As the replacement ratio of coal gangue fine powder increased, the calcium‐silicon ratio (Ca/Si) in the cement paste increased. The cement paste with a greater Ca/Si ratio exhibited an unstable structure, resulting in decreasing macroscopic concrete strength.
  c) Increasing the replacement ratio of CGP significantly hindered the hydration reaction and led to limited development of hydration products.
  d) Increasing the replacement ratio of CGP enhanced the volcanic ash effect (secondary hydration reaction), resulting in the formation of a small amount of hydration products that contributed to the strength. However, the contribution of the volcanic ash effect to the strength was far from sufficient to compensate for the loss of strength caused by the hindrance of the primary hydration reaction.
3) With increasing WCR, the intensity of the Ca(OH)_2_ diffraction peak decreased. When the WCR increased, the water content increased, leading to the dissolution and erosion of the hydration product Ca(OH)_2_. As a result, the structure of the cementitious paste became porous and loose. This was manifested macroscopically as a decrease in the strength of the concrete.

Due to the increase in the proportion of CGP and the water‐cement ratio, the strength of concrete decreases. Therefore, it is recommended that CGP‐RAC be used for non‐structural applications such as sidewalks and hollow wall panels. The CGP replacement rate should not exceed 20%, but the water‐cement ratio can be reduced appropriately to enhance the strength of the concrete. Compared to other forms of addition (coarse aggregates, fine aggregates, and gel materials), CGP has a greater impact on the performance of recycled concrete. However, its advantage lies in the ability to reduce the use of Portland cement, while simultaneously using recycled aggregates to replace natural aggregates, further reducing carbon emissions.

## Conflict of Interest

The authors declare no conflict of interest.

## Data Availability

The data that support the findings of this study are available on request from the corresponding author. The data are not publicly available due to privacy or ethical restrictions.
